# Ecosystem Models Can Predict the Consequences of Conservation Management Decisions

**DOI:** 10.1111/ele.70116

**Published:** 2025-04-18

**Authors:** Matthew S. Woodstock, Holden E. Harris

**Affiliations:** ^1^ Cooperative Institute for Marine and Atmospheric Studies, Rosenstiel School of Marine, Atmospheric, and Earth Science University of Miami Miami Florida USA; ^2^ NOAA Southeast Fisheries Science Center Miami Florida USA

**Keywords:** Community dynamics modeling, Decision‐support tools, Ecological forecasting, Ecology theory, Ecosystem modeling, Ecosystem‐based management, Natural resource management

## Abstract

Botelho *et al*. claimed that ecosystem models cannot make accurate population forecasts and should not be used in conservation management. We agree that producing accurate forecasts from ecosystem models is difficult, but assert that it has been achieved and their utility as a decision‐support tool is evident.

## Graphical abstract

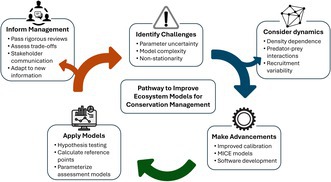

## 1 Technical Note

Ecosystem models integrate biological, environmental and socio‐economic data into a quantitative framework to forecast population dynamics, offering a decision‐support tool for conservation managers. Botelho et al. (2025) concluded that calibrated ecosystem models cannot make accurate predictions of management actions because of challenges surrounding parameter uncertainty, model complexity, non‐linearity and non‐stationarity. We fully agree that forecasting with ecosystem models is challenged by the combined influences of environmental, social, economic, density‐dependent and interspecific ecosystem components (Link et al. 2012; Storch et al. 2017). Indeed, reliance on ecosystem models (or any model) is not a substitute for careful adaptive management or adopting ensemble modelling approaches (Spence et al. 2021). However, the title, ‘calibrated ecosystem models ***cannot*** predict the consequences of conservation management decisions’ implies that ***none*** of these models are applicable in ***any*** conservation management forecast.

Quantitative population forecasting is just one management application, and a key point of potential confusion is that ‘ecosystem model’ is a broad term that includes a variety of objectives, algorithms and data needs. These may include single‐species models that integrate environmental covariates (Stock and Miller 2021) and biogeochemical models that resolve nutrient pools and low‐trophic level food webs (Daewel et al. 2019), end‐to‐end wholistic models, among others (Geary et al. 2020). The utility of an ecosystem model as a decision‐support tool is dependent on the available data and tools and the specific management situation.

Botelho et al. (2025) base much of their argument around the inability of mass‐balance Lotka‐Volterra predator–prey assumptions (i.e., predator consumption directly proportional to prey population size) to produce accurate population trends. Indeed, simple Lotka‐Volterra models cannot simulate the realistic predator–prey dynamics of the system (Hilborn and Walters 1992), as these equations assume predator–prey encounters are non‐random and are not limited by prey availability (Walters et al. 1997). The authors extrapolate the findings from one modelling experiment and state, ‘We believe that these results have broader implications for ecosystem models as a class’. Although the authors also ran simulations with indirect interactions, none of their calibrations included density‐dependent compensatory feedbacks. These are well known to moderate predator–prey interaction rates—for example, via satiation, handling time, prey hiding (Holling 1965) and foraging arena dynamics (Ahrens et al. 2012)—which were not modelled by Botelho et al. (2025). For this reason, ecosystem modelling methods generally calculate non‐linear, predator–prey interaction rates (e.g., Ecopath with Ecosim, Atlantis) or allow rates to emerge from model dynamics (e.g., OSMOSE; Shin and Cury (2001)).

A key criticism by Botelho et al. (2025) was that a high number of parameters undermines the reliability of ecosystem model predictions, regardless of the trophic functions used. While parameter uncertainty is a recognised and nuanced challenge in modelling complex systems (Fulton et al. 2003; Link 2010; Plagányi et al. 2014), technological and conceptual advances have been made. First, automated calibration approaches that fit ecosystem models to data sets with overfitting penalties exist (Scott et al. 2015; Oliveros‐Ramos et al. 2017; Pethybridge et al. 2019; Bentley et al. 2024). When implementing these, we agree with Botelho et al. that historical data series should be of sufficient duration and scale to represent the natural variability in the population, not wholly derived from synthetic data and not overfitted by the model. Second, ecosystem models of intermediate complexity have been applied to focus on a minimum number of functional groups needed to model dominant contributors for specific management decisions (Morello et al. 2014; Punt et al. 2016; Thorson et al. 2019; Chagaris et al. 2020). Such models can find a ‘sweet spot’ that balances issues of model complexity while concurrently diminishing model bias by incorporating environmental information and ecological dynamics (Collie et al. 2016).

Ecosystem models provide a key tool for managers to identify trade‐offs of alternative management decisions, define social and management objectives and explore the potential consequences of management decisions. The assertion that ecosystem models are incapable of forecasting, and the suggestion that this alone negates their management utility, ignores the field's progress in developing parsimonious models that have been implemented for management decisions or their use in incorporating environmental information into population models, which can increase predictive skill (Park et al. 2019; Tsai et al. 2024). To be used for conservation management decisions, rigorous credibility and quality control standards are needed (Kaplan and Marshall 2016; Olsen et al. 2016). For example, the US National Marine Fisheries Service applies a formal review process with extensive model documentation and workshops, whereby final decisions are made by a panel of independent experts (United States Office of Management and Budget 2004; Craig and Link 2023). Ecosystem models have passed such rigorous reviews for use in fisheries management (e.g., NEFSC 2008; Drew et al. 2021; Kaplan and Marshall 2016). Such models have been implemented in conjunction with traditional stock assessment models to produce improved population forecasts and tactical ecosystem‐based catch advice: for example, to set ecological reference points for forage fish harvest in the U.S. Atlantic (Anstead et al. 2021) and incorporate red tide impacts in Gulf of America gag grouper management (Vilas et al. 2023; SEDAR 72). Aside from improving quantitative forecasting capabilities, we implore discussion be directed towards considering *what management situations can ecosystem models perform well? What management scenarios require alternative methods?*

## Author Contributions

Both authors conceptualized the article. M.S.W. developed the initial draft and graphical abstract. Both authors contributed to revisions.

### Peer Review

The peer review history for this article is available at https://www.webofscience.com/api/gateway/wos/peer‐review/10.1111/ele.70116.

## Linked Articles

Calibrated ecosystem models cannot predict the consequences of conservation management decisions https://onlinelibrary.wiley.com/doi/10.1111/ele.70034

## Data Availability

The authors have nothing to report.
